# Genetic and morphological analyses reveal a complex biogeographic pattern in the endemic barbel populations of the southern Italian peninsula

**DOI:** 10.1002/ece3.5521

**Published:** 2019-08-29

**Authors:** Serena Zaccara, Silvia Quadroni, Vanessa De Santis, Isabella Vanetti, Antonella Carosi, Robert Britton, Massimo Lorenzoni

**Affiliations:** ^1^ Department of Theoretical and Applied Sciences University of Insubria Varese Italy; ^2^ Department of Chemistry, Biology and Biotechnology University of Perugia Perugia Italy; ^3^ Department of Life and Environmental Sciences Faculty of Science and Technology Bournemouth University Poole UK

**Keywords:** endemic barbels, hydrographic network, isolation, mitochondrial DNA, morphometric, southern Italy

## Abstract

The Italian peninsula is a biodiversity hotspot, with its freshwater fish fauna characterized by high levels of local endemism. Two endemic fluvio‐lacustrine fishes of the genus *Barbus* (barbel, family Cyprinidae) have allopatric distributions in the Tyrrhenian and Adriatic basins of Italy. *Barbus plebejus* inhabits the mid‐ to northern Adriatic basins, while *B. tyberinus* is widespread in all central‐northern basins draining into the Tyrrhenian Sea. For basins in Southern Italy draining into the southern parts of these seas, there remains a knowledge gap on their barbel populations due to no previous genetic and morphological studies, despite their apparent biogeographic isolation. Correspondingly, this study quantified the presence and distribution of barbels in the Adriatic and Tyrrhenian basins of Southern Italy through genetic and morphological analyses of 197 fish sampled across eight populations. Testing of how local isolation has influenced the evolution and persistence of these populations was completed by examining sequence variation at two mitochondrial loci (cytochrome *b* and D‐loop) and performing geometric morphometric analyses of body shape, plus measuring 11 morphometric and meristic characters. Phylogenetic and morphological analyses revealed the presence of two genetically distinct lineages that differed significantly from adjacent *B. tyberinus* and *B. plebejus* populations. These two new taxa, here described as SI1 and SI2 *Barbus* lineages, are highly structured and reflect a complex mosaic biogeographic pattern that is strongly associated with the underlying hydrographical scenarios of the basins. The geographic isolation of these basins thus has high evolutionary importance that has to be considered for maintaining endemism.

## Graphical abstract

In Southern Italy, apparent biogeographic isolation may have influenced barbel evolutionary structure. Through genetic and morphological analyses, we quantified the presence and distribution of barbels in Southern Italy describing two new taxa, highly structured and reflecting a complex mosaic biogeographic pattern that is strongly associated with the underlying hydrographical scenarios of the basins.

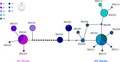

## 1 INTRODUCTION

The species richness of southern European freshwaters, including the peri‐Mediterranean area, is higher than in central and northern Europe, resulting in these freshwaters having high conservation value (De Figueroa, Fenoglio, & Sanchez‐Castillo, 2013). Biogeographically, the region is highly structured with, for example, the freshwater fish diversity between Southern Europe and Northern Africa comprising 23 different ecoregions (Abell et al., 2008; Geiger et al., 2014). Within this, more than 50 native freshwater fish are currently listed as present in the Italian peninsula (Bianco, 2014). The presence of a large number of rare taxa within this relatively small area was strongly influenced by geological and hydrological events during the glacial cycles of the Pleistocene (Bianco, 1995b, 1998; Hrbek & Meyer, 2003). These events resulted in the formation of three distinctive ichthyo‐geographic districts that are characterized by distinct evolutionary histories in species of the Cyprinidae family (Bianco, 1990, 1995a).

**Figure 1:**
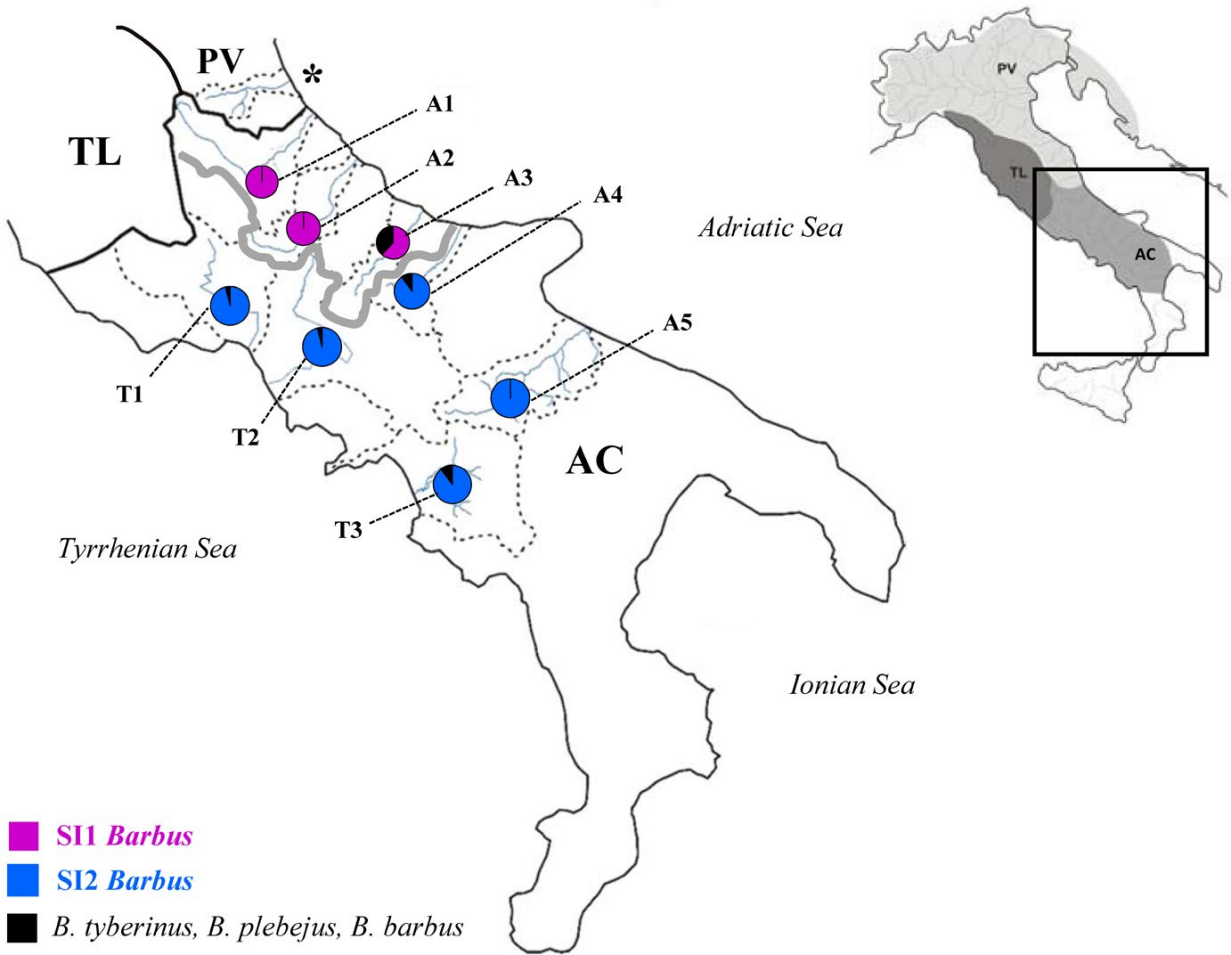
Map of sampling sites in South Italy, detailing SI1 and SI2 *Barbus* lineages boundary within the AC district. Biogeographic boundaries between the three Italian ichthyo‐geographic districts (PV = Padano‐Venetian; TL = Tuscano‐Latium; AC = Apulo‐Campano; sensu Bianco, 1990) are also reported in the insert. The colors of pie charts represent the frequency of phylogenetic lineages: black for *B. plebejus*, *B. tyberinus*, and *B. barbus*, while SI1 and SI2 *Barbus* lineages in purple and blue, respectively. Detailed frequencies are reported in Table 1. The asterisk indicates the Vomano basin

To date, fish biogeographic studies in the Italian peninsula have generally focused on the northern and central regions (e.g., Buonerba et al., 2015; Carosi, Ghetti, Forconi, & Lorenzoni, 2015; Livi et al., 2013; Marchetto, Zaccara, Muenzel, & Salzburger, 2010; Meraner et al., 2013; Stefani, Galli, Zaccara, & Crosa, 2004; Zaccara et al., 2019; Zaccara, Stefani, & Delmastro, 2007). These studies have centered on the Padano‐Venetian (PV) district of the Italian northeast region, including basins flowing into the upper and middle Adriatic Sea (north of the Vomano River in Abruzzo Region and the Krka River in Croatia), and on the Tuscano‐Latium (TL) district of central western region, including all basins draining into the middle Tyrrhenian Sea (Bianco, 1990, 1995a). Conversely, the Apulo‐Campano (AC) district of the southern region of Italy, which includes all basins flowing into southern Adriatic, southern Tyrrhenian, and Ionian seas (Bianco, 1990, 1995a; Figure 1), has received little research attention. For studies that have been completed, evidence suggests the AC district has long been isolated, and so might have been less influenced by lowered sea levels that occurred during Pleistocene period than basins further north (e.g., Bianco, 2014; Ketmaier et al., 2004), such as the paleo‐Po drainage (Bianco, 2014; Buonerba et al., 2015; Livi et al., 2013; Stefani et al., 2004; Zaccara et al., 2019).

Testing the evolutionary effects of the isolation of the southern Italian hydrographic basins, and the potential patterns and processes relating to vicariance events and local dispersal, can be completed using their cyprinid fish communities, as these generally show strong patterns of local endemism (Avise, 2000; Kottelat & Freyhof, 2007; Reyjol et al., 2007; Zardoya & Doadrio, 1999). Cyprinid fishes are widespread throughout all peri‐Mediterranean districts, but have limited capability of moving between hydrographic basins due to impassable watershed boundaries, coupled with low saline tolerances that result in coastal areas being effective barriers to their mixing. Among cyprinid fishes, barbels (species of the genus *Barbus*) have been used widely to study regional biogeography patterns and dynamic changes in continental and inland waters due to their marked diversity, wide distribution, and varied ecology (Buonerba et al., 2015; Gante, 2011). The genus *Barbus* includes species adapted to a variety of freshwater habitats, ranging from small mountain streams to large and slow‐flowing rivers and lakes (Kottelat & Freyhof, 2007).

In the Italian peninsula, three barbel species are considered endemic (Kottelat & Freyhof, 2007): common barbel *Barbus plebejus* Bonaparte, 1839; Tiber barbel *Barbus tyberinus* Bonaparte, 1839; and *Barbus caninus* Bonaparte, 1839. The habitat preferences of common and Tiber barbels are for larger, slower flowing rivers that are characterized by laminar flows and relatively warm temperatures (Kottelat & Freyhof, 2007). *Barbus plebejus* and *B. tyberinus* have an allopatric distribution in the Adriatic and Tyrrhenian basins, respectively (Buonerba et al., 2015; Zaccara et al., 2019). *Barbus plebejus* is widespread in the Adriatic basins (PV district), with an approximate southern limit of its range localized between the Tronto and Vomano rivers (Bianco, 1994, 2003a; Kottelat & Freyhof, 2007). Conversely, *B. tyberinus* is distributed in the main Tyrrhenian basins (Bianco, 2003b). *Barbus caninus* Bonaparte, 1839 is a small‐sized rheophilic barbel (total length up to c. 25 cm) that inhabits mountain brooks in the PV district (Kottelat & Freyhof, 2007; Tsigenopoulos & Berrebi, 2000). In recent studies, *B. plebejus* and *B. tyberinus* have been confirmed as distinct species based on genetic (Buonerba et al., 2015) and morphological differences (Lorenzoni et al., 2006; Zaccara et al., 2019), despite their similar fluvio‐lacustrine ecology.

To fill this considerable knowledge gap on the endemism of barbels in the AC district, the aim here was to test how local hydrographic history has influenced the evolution and persistence of the fluvio‐lacustrine barbels in the southern Italian peninsula. Mitochondrial sequence data and morphological analyses were applied to examine the extent of diversification of the barbels in the AC district compared with barbel populations in northern and central Italy. The results were then used to construct further hypotheses based on biogeographic scenarios that might have influenced patterns of endemism in the southern Adriatic and Tyrrhenian Sea hydrographical networks.

## 2 MATERIALS AND METHODS

### 2.1 Sampling

A total of 197 specimens of *Barbus* spp. were sampled in AC district between 2017 and 2018 with local authority permission. Fish were sampled from three sites in the Tyrrhenian basins and from five sites in the Adriatic basins. The Tyrrhenian sites were the basins Liri‐Garigliano (T1) and Volturno (T2), both close to TL district boundary, and Sele (T3) basin, located in the southern part. The Adriatic sites were in the Aterno‐Pescara (A1) basin that represents the first Adriatic drainage in AC district, and the Sangro (A2), Biferno (A3), Fortore (A4) up to Ofanto (A5) basins (see Table 1; Figure 1).

**Table 1:** Sampling site locations (expressed with ID code), watershed, river basin, and the number of individuals of each species sampled by site attributed through D‐loop mtDNA phylogenetic tree

ID code	Watershed	Basin	Latitude–Longitude	mtDNA *Barbus taxa*				
				SI1 *Barbus*	SI2 *Barbus*	*Barbus tyberinus*	*Barbus plebejus*	*Barbus barbus*
A1	Adriatic	Aterno‐Pescara	42°10′25.85″N−13°49′51.35″E	24				
A2		Sangro	42°05′29.76″N−14°34′75.82″E	23				
A3		Biferno	41°43′21.41″N−14°43′26.94″E	13			8	
A4		Fortore	41°33′13.20″N−14°52′33.92″E		27	3		
A5		Ofanto	41°07′39.23″N−15°54′62.24″E		20			
T1	Tyrrhenian	Liri‐Garigliano			25		1	
T2		Volturno	41°52′38.92″N−13°27′11.12″E		23			1
T3		Sele	41°58′72.53″N−14°16′20.98″E		26		3	

Note

The sampling site position, geographic coordinates, and barbel composition have been indicated also in Figure 1.

Sampling of the fish was completed using electric fishing. Captured specimens were removed from the water and then held in aerated tanks of water. Under general anesthesia (MS‐222), the fish were attributed to a species according to their phenotypic characteristics (e.g., colouration pattern, spot form and size, fin color; Kottelat & Freyhof, 2007; Lorenzoni et al., 2006), enabling recognition of the *B. tyberinus* phenotype as per Bianco (1995b). Each fish was then measured (fork length, nearest mm), and a biopsy of the anal fin was taken, preserved in 90% ethanol, and stored at 4°C for subsequent DNA extraction. For morphological analyses, fish were also photographed (left side) using a Nikon D300 camera (24–85 mm lens) positioned by means of a tripod on a table with a millimetric scale. The fish were then placed into another aerated water tank and, following their recovery to normal behavior, were released back into the river.

### 2.2 Molecular data

Total genomic DNA was extracted from all individuals using a proteinase K digestion, followed by sodium chloride extraction and ethanol precipitation (Aljanabi & Martinez, 1997). Two sets of primers were used to amplify mitochondrial control region (D‐loop) and cytochrome *b* (cyt *b*) gene (Livi et al., 2013). D‐loop sequences were obtained from the 197 individuals and used for all genetic analyses, while cyt *b* sequences were analyzed for a subsample of 26 fish, selected as a representative pool of the fish with specific D‐loop haplotypes. The mtDNA D‐loop fragment of 871 bp length was amplified using D‐loopsxF and D‐loopdxR (Antognazza, Andreou, Zaccara, & Britton, 2016; Rossi et al., 2013) primers pair, while cyt *b* gene using L15267 and H16461 (Briolay, Galtier, Brito, & Bouvet, 1998). Both PCR reactions were performed using Multiplex PCR kit (Qiagen) in 10 µl reaction volume containing approximately 10 ng of template DNA and 0.25 µM of each primer pair, using the same thermal cycle protocol (c.f. Zaccara et al., 2019). PCR products were purified using ExoSAP‐IT™ (USB) and directly sequenced by MACROGEN Inc (http://www.macrogen.org) using a 3730XL DNA Sequencer. All new haplotypes generated in this study were deposited in the GenBank database (Accession number MK728797–MK728821; MG718025–MG718026).

### 2.3 Phylogenetic analyses

Multiple alignments of all sequences were automatically carried out through ClustalW within Bioedit software (Hall, 1999), with polymorphic sites then checked manually. Identical sequences were collapsed into haplotypes in order to facilitate computational processes, as implemented in DnaSP v 5.0 (Librado & Rozas, 2009) software. Computation of mitochondrial phylogeny was performed independently for each gene on nonredundant haplotypes and on combined cyt *b* and D‐loop fragments dataset. For all phylogenetic analyses, two different phylogenetic inference methods were used as follows: maximum likelihood and Bayesian analyses. The former was conducted in GARLI v 2.0 (Bazinet, Zwickl, & Cummings, 2014; Zwickl, 2006) software, applying the specific setting for best evolutionary models. This was identified using Akaike's information criterion, as implemented in JModelTest v 2.1.10 (Darriba, Taboada, Doallo, & Posada, 2012): GTR + I (Lanave, Preparata, Sacone, & Serio, 1984; Rodriguez, Oliver, Marin, & Medina, 1990) and HKY85 (Hasegawa, Kishino, & Yano, 1985) for cyt *b* and D‐loop, respectively, and HKY85+I+G (Hasegawa et al., 1985) for the combined dataset. The GARLI tree searches were performed under the default settings. Support was assessed with 1,000 bootstrap replicates in GARLI, under the same settings as the best‐tree searches. The resulting bootstrap support values were mapped onto the maximum likelihood phylogeny using PAUP software (Swofford, 2002). Bayesian analyses were performed using four independent runs of Markov Montecarlo coupled chains of 4 × 10^6^ generations, each in order to estimate the posterior probability distribution, as implemented MrBayes v 3.1.2 (Ronquist et al., 2012) software. Topologies were sampled every 100 generations, and the majority‐rule consensus tree was estimated after discarding the first 25% of generations. For D‐loop, cyt *b*, and combined mitochondrial genes, *Luciobarbus graellsii* (JN049525 for cyt *b* and MG827110 for D‐loop, respectively) was used as an outgroup. The Cyt *b* and D‐loop sequences of *Barbus* species available in GenBank were included in the cyt *b* and D‐loop phylogenetic inferences: *B. barbus*, *B. plebejus*, and *B. tyberinus* (Buonerba et al., 2015; Meraner et al., 2013; Zaccara, Antognazza, Buonerba, Britton, & Crosa, 2014; Zaccara et al., 2019). To strengthen the cyt *b* phylogenetic tree, available sequences of rheophilic species (e.g., *B. caninus* and *B. balcanicus*) were also added (KC818238–KC818239 and KC818250–KC818251; Buonerba et al., 2015). Pairwise uncorrected p‐distances derived from mtDNA cyt *b* per lineages were estimated using PAUP software (Swofford, 2002) and used as a surrogate for levels of species divergence (Doadrio, Carmona, & Machordom, 2002).

### 2.4 Minimum spanning network, genetic diversity, and demography

A minimum spanning network was created from the multiple D‐loop sequences alignment produced in this study using a statistical parsimony criterion as implemented in PopART v 1.7 software (Leigh & Bryant, 2015). The levels of genetic variation within any new endemic lineages were then calculated by estimating nucleotide differences and haplotype diversity using DnaSP v 5.0 software. To visualize their historical demographic trends, mismatch analyses were performed, as implemented in Arlequin v 3.5 (Excoffier & Lischer, 2010) software, testing the sudden demographic expansion model through sum‐of‐squared deviation values (SSD) in a coalescent algorithm simulation over 1,000 pseudo‐replications with statistical significance (*p* < .05). To test the isolation between populations (within and between Tyrrhenian and Adriatic basins), population genetic differentiation was calculated using the fixation index Φ_ST_ (Weir & Cockerham, 1984) and its significance assessed (*p* < .05) by permuting haplotypes between populations 3,024 times, as implemented in Arlequin v 3.5.

### 2.5 Morphological data

**Figure 2:**
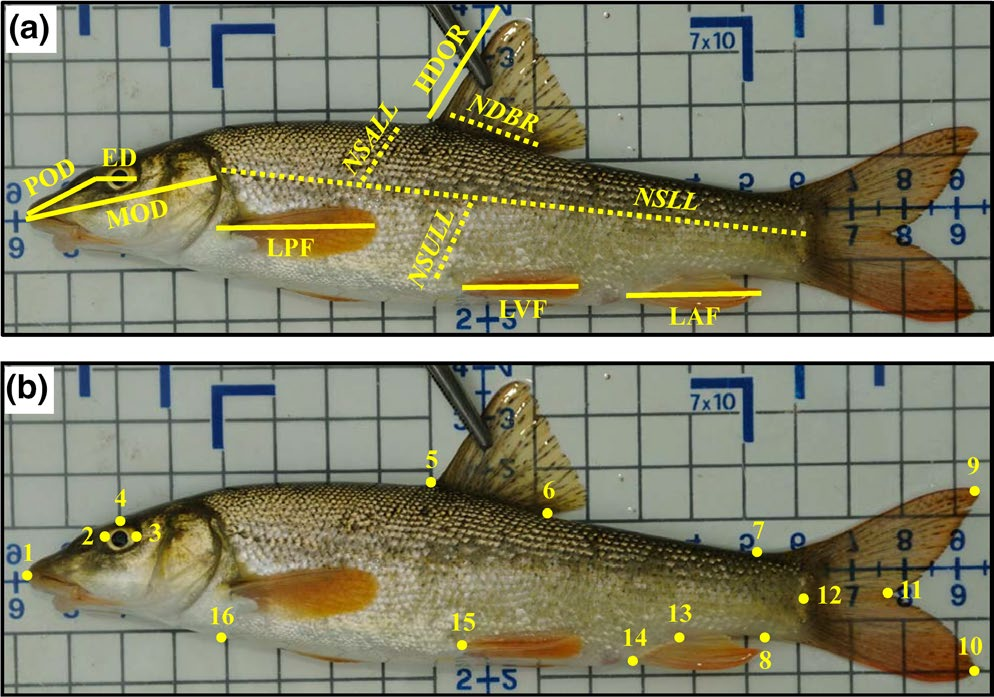
(a) Seven morphometric (ED, eye diameter; HDOR, height of the third dorsal fin ossified ray; LAF, length of anal fin; LPF, length of pectoral fin; LVF, length of ventral fin; MOD, mouth‐operculum distance; POD, preorbital distance) and four meristic traits (NDBR, the number of dorsal fin branched rays; NSLL, the number of scales on the lateral line, and on rows above—NSALL—and under—NSULL—the lateral line) considered for morphological analyses. (b) Position of the 16 landmarks used for body shape analysis: (1) anterior tip of snout, (2, 3) anterior and posterior end of the eye, (4) orthogonal projection on the dorsal profile of the eye center, (5, 6) anterior and posterior insertion of dorsal fin, (7, 8) anterior attachment of dorsal and ventral membrane of caudal fin, (9, 10) end of the upper and lower lobe of caudal fin, (11) “furca” of caudal fin, (12) base of middle caudal rays, (13, 14) posterior and anterior insertion of anal fin, (15) insertion of pelvic fin, and (16) orthogonal projection on the ventral profile of the (anterior) insertion of pectoral fin

The morphology of the barbel specimens was analyzed by measuring seven morphometric and four meristic traits as per Zaccara et al. (2019) (Figure 2a). Geometric morphometric analyses of body shape were performed by measurements of 16 landmarks (LMs) from the digital images within the R Geomorph function “digitize2d” (Adams, Collyer, & Kaliontzopoulou, 2018; Figure 2b). Attention was dedicated in positioning of caudal fin in order to include caudal fin LMs in the geometric morphometric analyses (9, 10, and 11; see Figure 2b), in agreement with Zaccara et al. (2019), obtaining results that were unchanged when caudal fin LMs were excluded. To strengthen the morphological differences between evolutionary barbel lineages, these data were combined with those from closely related taxa in central Italy (i.e., *B. tyberinus*, *B. plebejus*, and *B. barbus*; Zaccara et al., 2019). Nonshape variation, introduced through variation in position, orientation, and size, was mathematically removed using generalized procrustes analysis, as implemented in MorphoJ software (Klingenberg, 2011). Shape variations were then analyzed by canonical variate analyses (CVA). Mahalanobis distances were calculated using permutation tests (10,000 replicates). Morphometric traits were standardized to the overall mean standard length (Beacham, 1985) to reduce the effects of size and allometry. Pairwise comparison on morphological traits was then recorded between taxa and between populations by performing the analysis of variance (ANOVA) followed by the Tukey post hoc test. These analyses were carried out using PAST software (Hammer, Harper, & Ryan, 2001).

## 3 RESULTS

### 3.1 Multiple alignments and phylogeny

Across the 197 barbels, 26 haplotypes were identified in the 871 bp length of the multiple D‐loop alignment, of which 19 were new and deposited in GenBank (under Accession numbers: MK728797–MK728815) as detailed in Table 2. There were 26 variable nucleotide positions detected, of which eight were singletons and 18 were parsimony informative sites. Partial cyt *b* sequences of 714 bp length were obtained from each new D‐loop haplotype; in the multiple alignment, 22 variable sites (21 singletons and one parsimony site) were scored and seven new haplotypes detected (GenBank accession numbers: MK728816–MK72821; MG718025–MG718026, see Table 2).

**Table 2:** Haplotype distribution and frequencies of D‐loop mtDNA fragment (871 bp length) of 181 barbels belonging to SI1 and SI2 *Barbus* lineages

*Barbus* lineages	D‐loop haplotype	Adriatic basins					Tyrrhenian basins			Tot	D‐loop GB acc. no.	Cyt *b* GB acc. no.
		A1	A2	A3	A4	A5	T1	T2	T3			
SI1	BSI101	22	12							34	MK728797	MG718025
	BSI102			13						13	MK728798	MK728816
	BSI103		11							11	MK728799	MG718025
	BSI104	1								1	MK728800	MG718026
	BSI105	1								1	MK728801	MG718025
SI2	BSI201					15		12	22	49	MK728802	MK728817
	BSI202				22					22	MK728808	MK728819
	BSI203						12		1	13	MK728809	MK728821
	BSI204						13			13	MK728810	MK728817
	BSI205							3	1	4	MK728811	MK728817
	BSI206					2		1		3	MK728812	MK728817
	BSI207							2	1	3	MK728813	MK728817
	BSI208							1		1	MK728814	MK728817
	BSI209					3		1	1	5	MK728815	MK728817
	BSI210							1		1	MK728803	MK728817
	BSI211							1		1	MK728804	MK728820
	BSI212							1		1	MK728805	MK728817
	BSI213				4					4	MK728806	MK728819
	BSI214				1					1	MK728807	MK728819

Note

All new haplotypes were deposited in GenBank under accession numbers MK728797–MK728815. For each D‐loop haplotype, the corresponding GenBank accession number of new cyt *b* haplotype is reported.

**Figure 3:**
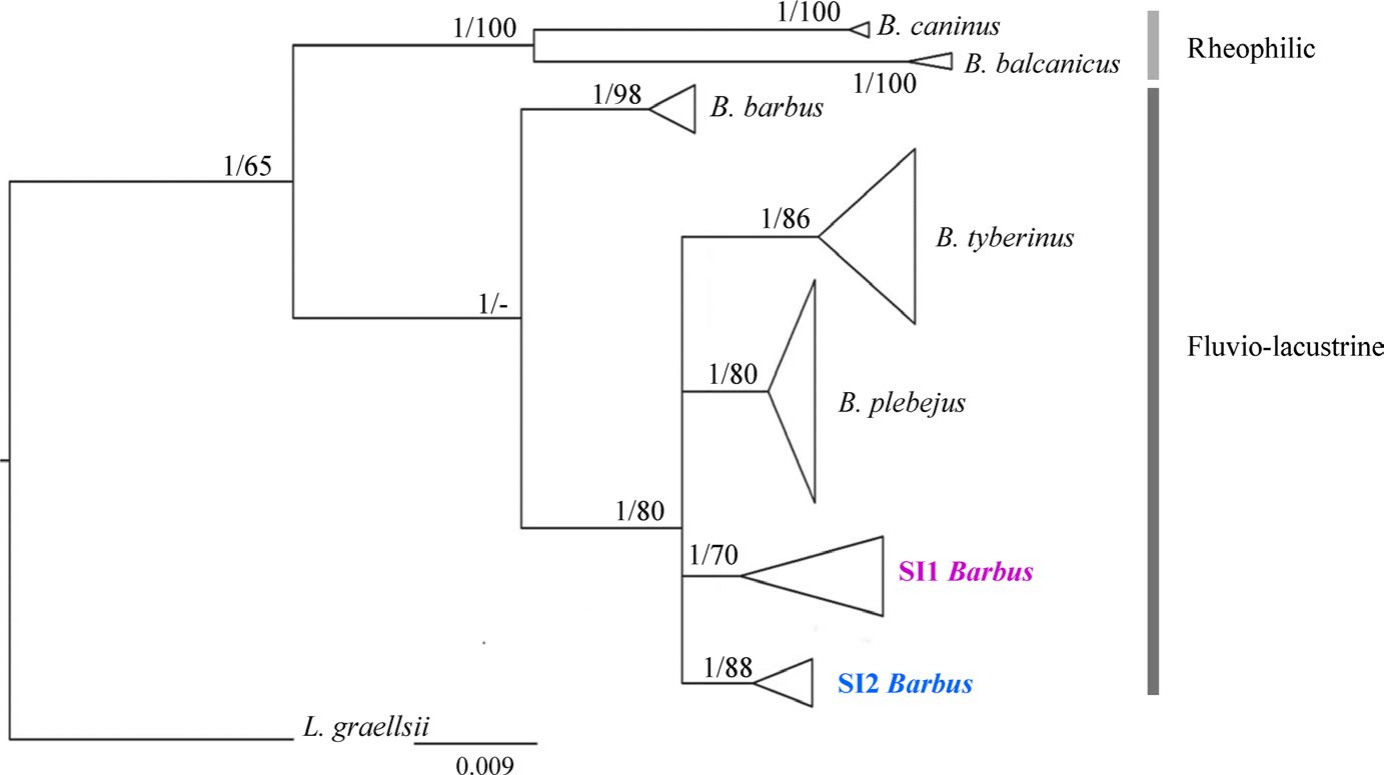
Phylogenetic tree built upon cyt *b* sequences (714 bp length). Statistic support is given and expressed both as posterior probability and bootstrap values. The tree was rooted on *Luciobarbus graellsii* (GenBank accession number JN049525)

Maximum likelihood and Bayesian analysis of the mitochondrial cyt *b* sequences separated out the all fluvio‐lacustrine and rheophilic *Barbus* (*B. barbus*, *B. plebejus*, *B. tyberinus*, *B. caninus*, and *B. balcanicus*) species well, but as they did not clearly resolve the evolutionary relationships, they showed unresolved polytomy (Figure 3). Within the fluvio‐lacustrine species cluster, D‐loop and combined phylogenetic trees (Figure S1 and S2) were congruent, clustering 16 fish as *B. barbus*, *B. plebejus*, *B. tyberinus*, and, for the first time, two new *Barbus* monophyletic lineages in the AC district. These lineages are named here as “South Italy 1” (SI1) and “South Italy 2” (SI2) *Barbus* lineages. In the D‐loop phylogenetic tree, the haplotypes recorded in Vomano River (c.f. Zaccara et al., 2019) were clustered in SI1 *Barbus* lineage.

The uncorrected p‐distance values calculated on the cyt *b* sequences between the SI1 and SI2 *Barbus* lineages and European (*B. barbus*) barbel were 3.9% and 3.6%, respectively. It is noteworthy that SI *Barbus* lineages were more similar to *B. plebejus* (1.5%–1.8%) than to *B. tyberinus* (2.1%–2.4%) and that the inter‐lineage uncorrected p‐distance between SI1 and SI2 *Barbus* lineages (1.7%) was in a middle position (Table 3).

**Table 3:** Uncorrected p‐distances (expressed as percentage) calculated on 714 bp length of cyt *b* mtDNA for five fluvio‐lacustrine *Barbus* lineages (*B. barbus*, *B. plebejus*, *B. tyberinus*, SI1, and SI2 *Barbus*; see Figure 1)

Lineages	*B. barbus*	*B. plebejus*	*B. tyberinus*	SI1 *Barbus*	SI2 *Barbus*
*B. barbus*	0.23 ± 0.11				
*B. plebejus*	3.87 ± 0.14	0.29 ± 0.1			
*B. tyberinus*	4.16 ± 0.23	2.13 ± 0. 20	0.39 ± 0.17		
SI1 *Barbus*	3.86 ± 0.43	1.82 ± 0.43	2.41 ± 0.41	0.87 ± 0.53	
SI2 *Barbus*	3.55 ± 0.19	1.52 ± 0.18	2.10 ± 0.20	1.69 ± 0.36	0.21 ± 0.15

### 3.2 Networks, genetic diversity, and demography of South Italy lineages

**Figure 4:**
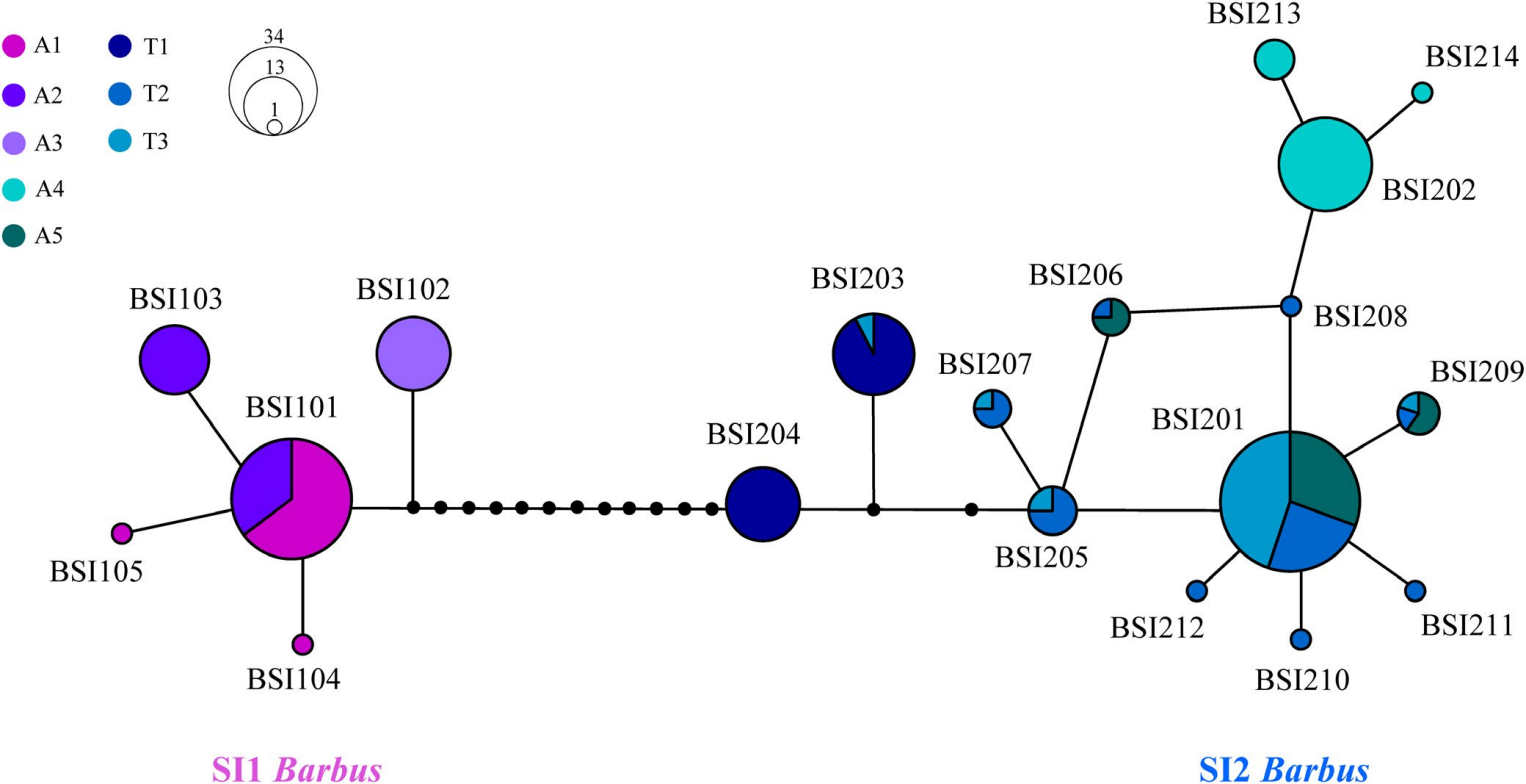
Parsimony network obtained from D‐loop sequences (871 bp length) belonging to South Italy *Barbus* lineages (SI1 and SI2; see Table 2). Circle size is proportional to haplotype frequencies. Colors indicate Adriatic (A1 = Aterno‐Pescara; A2 = Sangro; A3 = Biferno; A4 = Fortore; A5 = Ofanto) and Tyrrhenian (T1 = Liri‐Garigliano; T2 = Volturno; T3 = Sele) populations

In the network analyses of the complete mitochondrial D‐loop dataset, the SI1 and SI2 *Barbus* lineages (*N* = 181) were linked by more than 13 mutational steps and revealed some distinct patterns. The SI1 *Barbus* lineage (*N* = 60) was composed by five new haplotypes that were connected by up to seven mutational steps, with the most frequent BSI01 positioned in the middle of the radiation (Figure 4). The SI2 *Barbus* lineage (*N* = 121) showed a larger number of haplotypes (i.e., 14), with the two most frequent haplotypes (BSI201 and BSI202) separated by four mutational steps (Figure 4). Genetic diversity of the SI1 and SI2 *Barbus* lineages had values of nucleotide diversity (*π*) of 0.001 and 0.003, and haplotype diversity (H) of 0.61 and 0.78, respectively. The mismatch distribution analyses do not support a sudden expansion model for both lineages (SSD = 0.007, *p* = .58 in SI1 and SSD = 0.0283, *p* = .22 in SI2), as they revealed multiwave trends (Figure S3).

### 3.3 Haplotype distribution and population structure

In the AC district, the SI1 and SI2 *Barbus* lineages showed an allopatric distribution. The SI1 *Barbus* lineage was recorded in middle Adriatic basins (from A1 up to A3), whereas the SI2 *Barbus* lineage was present both in the three middle Tyrrhenian basins (T1, T2, and T3) and in the two most southern Adriatic basins (A4 and A5; see Figure 1 and Table 1). Genetic differentiation between the SI1 *Barbus* lineage of the three middle Adriatic populations revealed high genetic structure, with significant ф_ST_ values over 0.39 (*p* < .01; Table S1). Genetic differentiation was also recorded between the five populations of the SI2 *Barbus* lineage, with ф_ST_ values ranging between 0.71 and 0.89 (*p* < .01). Among the AC district barbel populations, only the A5, T2, and T3 populations were dominated by the BSI201 haplotype (SI2 *Barbus* lineage; Figure 4) and did not show significant differentiation (*p* > .05; Table S1).

### 3.4 Morphological pattern among lineages and among populations

**Figure 5:**
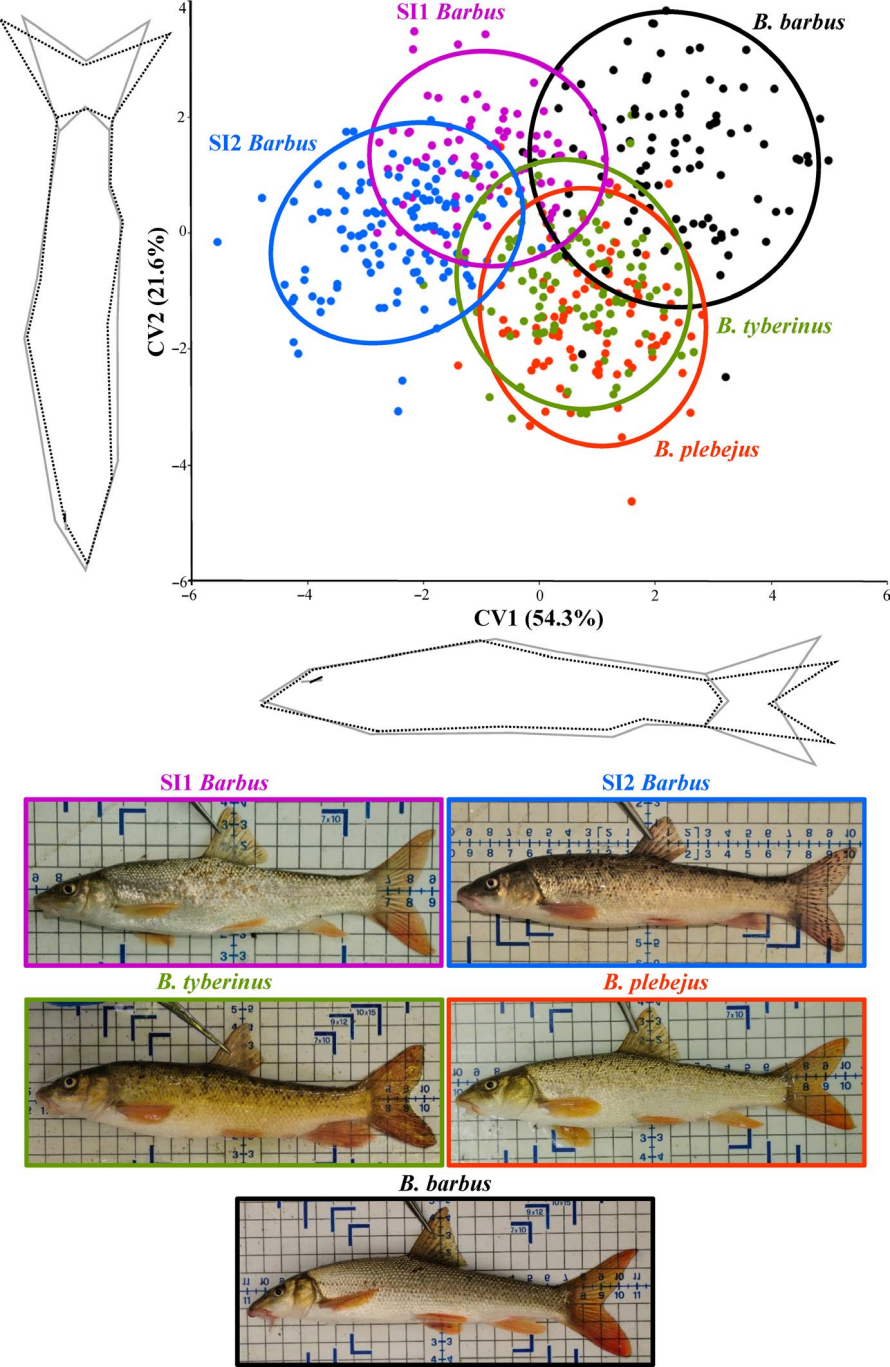
Canonical variate analysis (CVA) output of the body shape comparison between the *Barbus* lineages detected in this study (SI1 and SI2) and *B. tyberinus*, *B. plebejus*, and *B. barbus* species from Zaccara et al. (2019). Wireframe graphs indicate the shape changes along each axis (from gray to dashed black). A sample photograph is shown for each taxon

The geometric morphometric analyses of the CVA plot revealed there was partial visual separation in body shape morphology in the two SI *Barbus* lineages (Figure 5). This was supported by Mahalanobis distances that ranged between 3.26 and 4.96 (all *p* < .05). Variations along the CV1 (54%) were mainly associated with the eye diameter, the depth of the posterior body, and the shape of the caudal fin; those along the CV2 (22%) were mainly associated with the overall fish body shape. The SI1 and SI2 *Barbus* lineages were partially separated from each other along both axes, as also indicated by the Mahalanobis distance value (MD = 3.27). Comparisons with the other two Italian *Barbus* species revealed the SI1 *Barbus* lineage had a higher overlapping position with *B. tyberinus* (MD = 3.26) than with *B. plebejus* (MD = 3.59). The SI2 *Barbus* lineage was more separated from both *B. tyberinus* (MD = 3.58) and *B. plebejus* (MD = 4.01). Both SI *Barbus* lineages showed the highest Mahalanobis distance values against *B. barbus* (MD = 4.09 and 4.96 with SI1 and SI2 *Barbus* lineages, respectively), and, in the case of SI2 *Barbus* lineage, a complete separation with the exotic *B. barbus* was observed in the CVA plot.

The ANOVA results (Table 4) and Tukey post hoc test for the pairwise comparison on morphological traits (Table S2) revealed statistical distinction (*p* < .05) between the SI1 and SI2 *Barbus* lineages for all the analyzed traits, except for the number of dorsal fin branched rays and the number of scales on the lateral line. Both lineages had values of the latter character that were not statistically different from *B. tyberinus* (*p* > .05). Moreover, no significant differences were recorded between SI1 *Barbus* lineage and *B. tyberinus* for any of the morphometric traits (*p* > .05), except for the height of the third dorsal fin ray (*p* < .05). The SI2 *Barbus* lineage was not statistically different from *B. plebejus* (*p* > .05), both for all the morphometric traits and for the number of dorsal fin branched rays.

**Table 4:** List of morphometric and meristic traits, number of individuals (*N*), mean (±standard deviation), and minimum–maximum range for *Barbus* groups detected in this study and by Zaccara et al. (2019)

		SI1 *Barbus* *N* = 85	SI2 *Barbus* *N* = 121	*B. tyberinus* *N* = 107	*B. plebejus* *N* = 96	*B. barbus* *N* = 96	ANOVA *F*
Morphometric traits (cm)							
Eye diameter	ED	0.67 ± 0.11 (0.46–1.03)	0.62 ± 0.10 (0.41–0.91)	0.66 ± 0.12 (0.36–0.95)	0.62 ± 0.13 (0.37–1.02)	0.73 ± 0.14 (0.48–1.14)	13.9
Preorbital distance	POD	1.53 ± 0.40 (0.78–2.86)	1.22 ± 0.33 (0.57–2.39)	1.50 ± 0.46 (0.60–2.71)	1.33 ± 0.45 (0.55–2.84)	1.78 ± 0.48 (0.93–3.03)	25.8
Mouth‐operculum distance	MOD	3.69 ± 0.79 (2.37–6.31)	3.15 ± 0.66 (1.88–5.14)	3.62 ± 0.82 (1.83–5.85)	3.31 ± 0.89 (1.70–6.39)	4.03 ± 0.89 (2.38–6.12)	19.0
Length of pectoral fin	LPF	3.07 ± 0.68 (1.68–5.60)	2.58 ± 0.57 (1.35–4.02)	2.87 ± 0.68 (1.07–4.68)	2.59 ± 0.83 (1.12–5.22)	3.29 ± 0.81 (1.86–5.30)	18.6
Length of ventral fin	LVF	2.36 ± 0.56 (1.17–4.17)	1.97 ± 0.43 (1.01–3.04)	2.22 ± 0.56 (1.04–3.81)	2.02 ± 0.62 (0.88–4.08)	2.71 ± 0.69 (1.44–4.49)	27.5
Length of anal fin	LAF	2.77 ± 0.73 (1.30–5.18)	2.30 ± 0.67 (1.23–4.21)	2.79 ± 0.93 (1.18–5.20)	2.37 ± 0.89 (1.09–5.93)	2.99 ± 0.72 (1.65–4.92)	14.4
Height of the third dorsal fin ossified ray	HDOR	2.03 ± 0.52 (1.13–3.74)	1.62 ± 0.37 (0.89–2.69)	1.83 ± 0.41 (1.01–3.03)	1.66 ± 0.54 (0.67–3.33)	2.10 ± 0.50 (1.15–3.54)	21.1
Meristic traits							
Number of dorsal fin branched rays	NDBR	7.9 ± 0.4 (7–9)	8.0 ± 0.3 (7–9)	8.1 ± 0.3 (7–9)	7.8 ± 0.5 (7–9)	8.1 ± 0.3 (7–9)	7.4
Number of scales on the lateral line	NSLL	55.8 ± 4.1 (50–70)	55.3 ± 2.8 (49–62)	56.0 ± 3.5 (50–66)	62.6 ± 3.8 (53–71)	56.9 ± 3.5 (49–68)	70.7
Number of scales above the lateral line	NSALL	11.1 ± 1.1 (9–14)	11.7 ± 1.1 (9–15)	12.2 ± 1.3 (10–16)	13.4 ± 1.1 (10–16)	12.2 ± 1.0 (10–15)	55.3
Number of scales under the lateral line	NSULL	7.9 ± 0.8 (6–10)	8.7 ± 0.8 (7–11)	8.5 ± 1.1 (6–13)	9.3 ± 1.0 (7–12)	8.4 ± 0.8 (7–10)	30.9

Note

Data of morphometric traits were transformed according to Beacham (1985) formula. ANOVA results (*F*) showing differences among the five *Barbus* groups are also reported; all *p*‐values were <.001.

**Figure 6:**
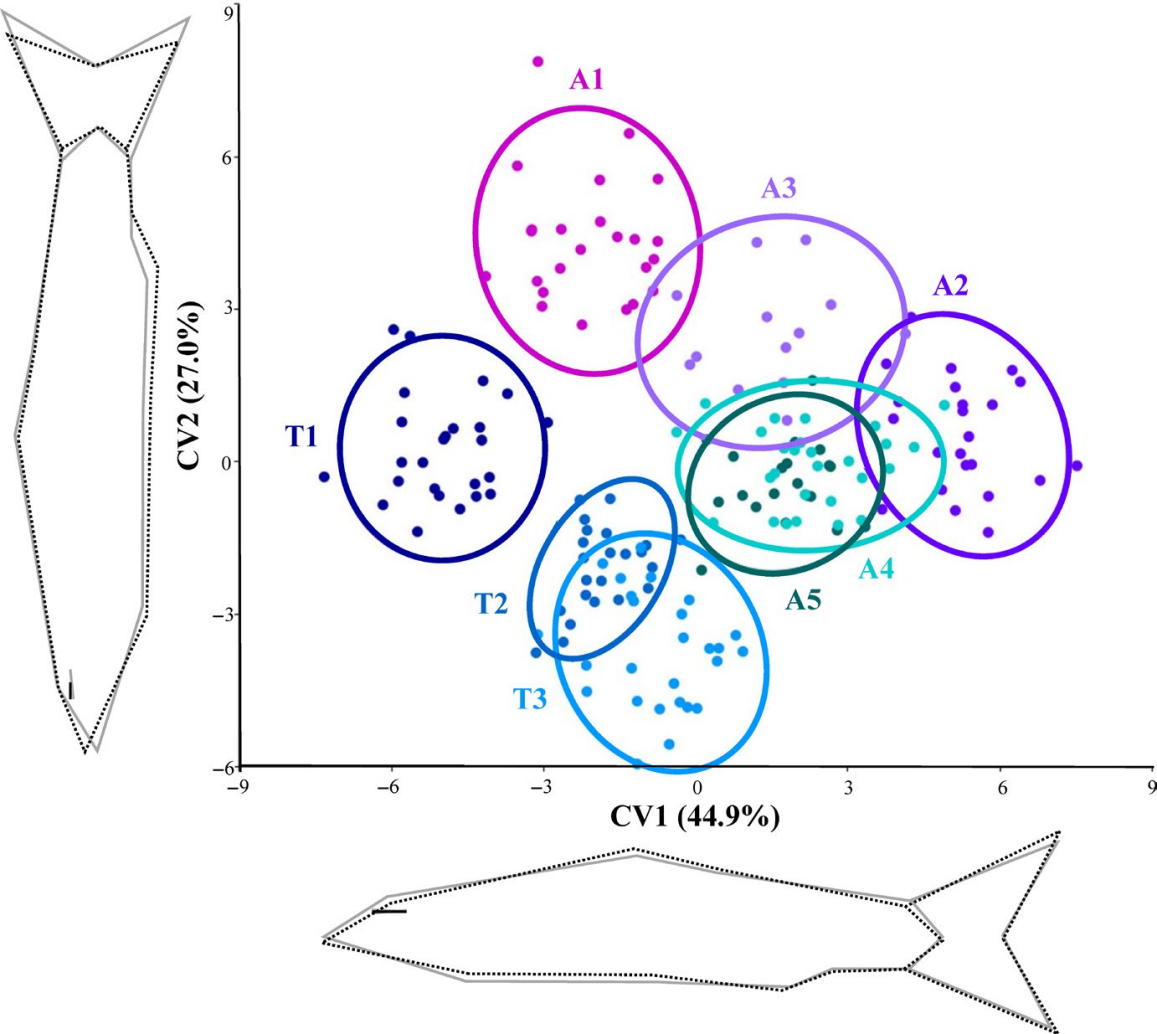
Canonical variate analysis (CVA) output of the body shape comparison between the eight populations of *Barbus* considered in the present study (see Figure 1). Wireframe graphs indicate the shape changes along each axis (from gray to dashed black)

Although the ANOVA results did not indicate relevant morphological differences among the barbel populations in southern Italy (most *p* > .05), the geometric morphometric analyses of the CVA plot indicated some visual separation (i.e., CV1 = 45% and CV2 = 27%; Figure 6). The barbel populations from the Tyrrhenian basins (T1, T2 and T3) were localized in the III quadrant of the CVA plot, while the Adriatic populations were in the I and II quadrants. Differences associated with the eye, and the anal and caudal fins, were detected along the CV2 axes that partially separated populations that were attributed to the SI1 *Barbus* lineage (A1, A2, and A3) from those attributed to the SI2 *Barbus* lineage (A4, A5, T1, T2, and T3). The minimum Mahalanobis distance (MD = 3.95) was recorded between the T2 and T3 populations, belonging to two contiguous Tyrrhenian basins, while the maximum value (MD = 10.50) was found between T1 and A2 populations (Table S3), inhabiting two basins located at similar latitude but on the opposite sides of the Italian peninsula (Figure 1).

## 4 DISCUSSION

Through the combined analyses of phylogeny, population genetic structure, distribution and characterization of morphological variability, the results revealed the first evidence for two allopatric *Barbus* evolutionary lineages in the AC district of Southern Italy that were also characterized by distinct morphotypes. These results raise a number of questions relating to their biogeography and their genetic and morphological differences.

Regarding their biogeography, their genetic and morphological variations may reflect the hydrographic and landscape evolution. The phylogenetic analyses revealed the existence of two new lineages that were only partially identifiable in the field and are considered in the literature as the *B. tyberinus* phenotype (Bianco, 2014). Furthermore, the allopatric distribution of the two new fluvio‐lacustrine barbel taxa (SI1 and SI2 *Barbus*) confirms the complex mosaic pattern recorded across the north and central Italian peninsula, where the allopatric origins and dispersion routes of the species have been primarily influenced by distinct historical events (Buonerba et al., 2015; Zaccara et al., 2019). In the north‐western Adriatic basins (PV district), the widespread distribution of *B. plebejus* occurred during the glacial cycles that promoted low sea level and low river connections (Buonerba et al., 2015; Meraner et al., 2013). The extended paleo‐Po basin reached the meso‐Adriatic ditch in the central Adriatic Sea (Bianco, 1990), joining rivers of the two Adriatic slopes (c.f. Italian and Balkan peninsula), and resulted in wide genetic admixture of *B. plebejus* (Bianco, 2014; Buonerba et al., 2015; Meraner et al., 2013). In the upper‐middle Tyrrhenian basins (TL district), fluvial connection within the rivers systems occurred due to the considerable extension of the hydrographic network along mountain and high hill environments, with this enabling more effective upstream colonization and widespread distribution of *B. tyberinus* (Carosi, Ghetti, La Porta, & Lorenzoni, 2017; Lorenzoni et al., 2006; Zaccara et al., 2019) up to the Liri‐Garigliano basin (T1) where the SI2 *Barbus* lineage was recorded for the first time. The allopatric distribution of these two species confirms there were specific biogeographic boundaries between districts along the Tyrrhenian and Adriatic slopes, constituted by the Rivers Liri and Vomano (see Figure 1), respectively. This biogeographic scenario has been demonstrated for more vicarious species, such as Volturno spined loach (*Cobitis zanandreai* Cavicchioli, 1965) and Italian bleak (*Alburnus albidus* Costa, 1838; Kottelat & Freyhof, 2007). The causes of this biogeographic split may be related to local differences in low sea level drainage patterns, although differences in habitats and in biotic interactions might also have been involved.

The results of the population genetic structure have also demonstrated a nonhomogeneous history in the AC basins, showing the presence of unexpected biogeographic boundary that crossed the Apennine watershed. Across the Italian peninsula, the mosaic biogeographic pattern of the genus *Barbus* was likely to be associated with the differing hydrographic structure of the basins. For example, the SI1 *Barbus* lineage appeared to originate and only be maintained in basins A1 to A3 (Pescara River up to Biferno River of the middle Adriatic). These basins were not part of the paleo‐Po expansion (Bianco, 1990), and so they remained isolated from the widespread dispersion of *B. plebejus* that occurred in the upper Adriatic basins (c.f. PV district). Within this restricted area, the SI1 *Barbus* lineage had high levels of genetic variability and was thus highly structured. These results suggest that climatic, hydrological, and geological factors probably shaped their local isolation and did not result in dispersion events via temporary connections (Forneris, Merati, Pascale, Perosino, & Tribaudino, 2016). Although the hydrogeographic layout of the AC region is congruent with the current topographic and geological pattern, the main distribution of watercourses has also been influenced by its lithological structure from previous geomorphological stages (Amato, Cinque, & Santangelo, 1995). Current knowledge on the geomorphological evolution of the southern Apennine chain has shown an asymmetric profile of the watershed line, with a retreat of the Tyrrhenian side and progression of the Adriatic side (Brancaccio & Cinque, 1992; Brancaccio et al., 1991). The temporary change in the draining path occurred between Sele (T3) and Ofanto (A5) basins, promoted by temporary river capture events or transitory mountain lakes, that might help explain the actual distribution of the SI2 *Barbus* lineage in both the southern Tyrrhenian basins (from T1 to T3; i.e., from Liri‐Garigliano to Sele basins) and the southern Adriatic basins (A4 and A5; i.e., Fortore and Ofanto basins; Alvarez, 1999), as also reflected by the absence of genetic structure.

Regarding the congruence of the genetic and morphological data, these Italian fluvio‐lacustrine barbels, representing a complex of cryptic species, were only partially identifiable by morphology, with their morphological and molecular divergence not always well correlated across the species (Bianco, 1995b; Kottelat & Freyhof, 2007; Livi et al., 2013; Lorenzoni et al., 2006; Zaccara et al., 2019). Despite this lack of congruence between the genetic and morphological approaches, there was nevertheless some significant correlation between evolutionary lineages and body shape. The two SI *Barbus* lineages were significantly differentiated from each other for all morphological traits, except for the number of dorsal fin branched rays and the number of scales on the lateral line, as per Antal et al. (2016). Moreover, looking at the dimension of the eye and at the caudal fin lobes, the barbel populations could be morphologically differentiated.

In conclusion, within the hydrogeographic units of the AC district of Southern Italy, there is high genetic structure in the barbel populations that can be related to the isolation of the basins, resulting in very limited gene flow between them. The limitation in dispersion was due to minimal river capture events in the upstream part of the basins that, due to their typically Mediterranean regime, are characterized by low discharge, and thus, the fish were unable to mix due to insurmountable geographical barriers. Consequently, the AC district can be considered as unique in relation to the biogeography of their endemic barbel populations, with their geographic and hydrological isolation from basins further north being important in this. These results emphasize that, across this district, the evolutionary processes of the endemic barbels have favoured a mosaic pattern, although it is suggested that this requires further work by use of an enlarged dataset, including studies on other freshwater taxa. Although we recorded a limited presence of *B. barbus*, *B. tyberinus*, and *B. plebejus* fish in the AC district, subsequent anthropic manipulation and translocations could still cause genetic admixture (i.e., hybridization) between *Barbus* species in future. If this happens, it is likely to remain undetected along this complex of cryptic species and will potentially lead to the loss of local endemism. Consequently, these results highlight the necessity for any fish and fishery management programmes in this region to recognize the inherently high conservation value of these endemic barbels and avoid undesirable mixing with other barbels through, for example, fish stocking exercises.

## CONFLICT OF INTEREST

None declared.

## AUTHORS' CONTRIBUTIONS

SZ, ML, SQ, and AC conceived the ideas and designed the methodology; ML and AC collected the data; SQ analyzed the photographs and performed morphological analysis; IV and VDS worked in laboratory and analyzed the data; and all authors contributed critically to the drafts and gave final approval for publication.

## Data Availability

All new haplotypes produced in this study have been deposed in GenBank under accession numbers: MK728797–MK72821; MG718025–MG718026.
