# Supplementary material for: Genetic and morphological analyses reveal a complex biogeographic pattern in the endemic barbel populations of the southern Italian peninsula
Source: Ecol Evol. 2019 Aug 29;9(18):10185–97. doi: 10.1002/ece3.5521 (PMC6787835; doi:10.1002/ece3.5521)
Supplement: Supplementary file 1 [file ECE3-9-10185-s001.docx]

**Supporting Information TABLES (S1-S3)**

**TABLE S1** Pairwise ф_ST_ values calculated for South Italy populations collected in Adriatic (A1 up to A5) and in Tyrrhenian (T1 up to T3) basins (see Figure 1). In bold significant values (p < 0.01).

|  | **SI1 *Barbus* lineage** | | |  | **SI2 *Barbus* lineage** | | | | |
| --- | --- | --- | --- | --- | --- | --- | --- | --- | --- |
| **ID pop** | A1 | A2 | A3 |  | A4 | A5 | T1 | T2 | T3 |
| A1 |  |  |  |  |  |  |  |  |  |
| A2 | **0.39** |  |  |  |  |  |  |  |  |
| A3 | **0.95** | **0.87** |  |  |  |  |  |  |  |
| A4 | **0.99** | **0.98** | **0.99** |  |  |  |  |  |  |
| A5 | **0.98** | **0.97** | **0.98** |  | **0.80** |  |  |  |  |
| T1 | **0.96** | **0.95** | **0.96** |  | **0.89** | **0.79** |  |  |  |
| T2 | **0.96** | **0.95** | **0.95** |  | **0.71** | 0.01 | **0.72** |  |  |
| T3 | **0.98** | **0.97** | **0.98** |  | **0.81** | 0.00 | **0.79** | 0.01 |  |

**TABLE S2** Results of the Tukey *post-hoc* test (Q and p values, the latter within brackets) for the pairwise comparison of the morphological traits (see Figure 2A) between the SI1 and SI2 *Barbus* lineages *versus* the other *Barbus* groups (see Table 4). In bold significant values (p < 0.05).

|  | **ED** | **POD** | **MOD** | **LPF** | **LVF** | **LAF** | **HDOR** | **NDBR** | **NSLL** | **NSALL** | **NSULL** |
| --- | --- | --- | --- | --- | --- | --- | --- | --- | --- | --- | --- |
| **SI1 *Barbus vs***  **SI2 *Barbus*** | **4.49**  **(0.013)** | **7.12**  **(<0.001)** | **6.75**  **(<0.001)** | **6.82**  **(<0.001)** | **6.86**  **(<0.001)** | **5.84**  **(<0.001)** | **8.75**  **(<0.001)** | 1.22  (0.912) | 1.41  (0.856) | **5.11**  **(0.003)** | **8.48**  **(<0.001)** |
| **SI1 *Barbus vs***  ***B. tyberinus*** | 0.77  (0.983) | 0.57  (0.994) | 0.88  (0.972) | 2.82  (0.270) | 2.54  (0.376) | 0.30  (1.000) | **4.36**  **(0.018)** | **4.34**  **(0.019)** | 0.66  (0.990) | **10.00**  **(<0.001)** | **6.91**  **(<0.001)** |
| **SI1 *Barbus vs***  ***B. plebejus*** | **4.50**  **(0.013)** | **4.67**  **(0.008)** | **4.66**  **(0.009)** | **6.62**  **(<0.001)** | **6.00**  **(<0.001)** | **4.96**  **(0.004)** | **7.95**  **(<0.001)** | 2.35  (0.459) | **18.85**  **(<0.001)** | **20.45**  **(<0.001)** | **15.84**  **(<0.001)** |
| **SI1 *Barbus vs***  ***B. barbus*** | **4.42**  **(0.015)** | **5.86**  **(<0.001)** | **4.19**  **(0.026)** | 3.15  (0.169) | **6.04**  **(<0.001)** | 2.83  (0.265) | 1.39  (0.865) | 3.83  (0.052) | 2.97  (0.221) | **10.01**  **(<0.001)** | **5.31**  **(0.002)** |
| **SI2 *Barbus vs***  ***B. tyberinus*** | 3.72  (0.065) | **6.55**  **(<0.001)** | **5.87**  **(<0.001)** | **4.00**  **(0.037)** | **4.32**  **(0.019)** | **6.15**  **(<0.001)** | **4.39**  **(0.016)** | 3.12  (0.177) | 2.07  (0.585) | **4.90**  **(0.005)** | 1.58  (0.799) |
| **SI2 *Barbus vs***  ***B. plebejus*** | 0.02  (1.000) | 2.45  (0.415) | 2.09  (0.576) | 0.20  (1.000) | 0.86  (0.974) | 0.88  (0.972) | 0.80  (0.980) | 3.56  (0.086) | **20.26**  **(<0.001)** | **15.34**  **(<0.001)** | **7.36**  **(<0.001)** |
| **SI2 *Barbus vs***  ***B. barbus*** | **8.91**  **(<0.001)** | **12.98**  **(<0.001)** | **10.94**  **(<0.001)** | **9.97**  **(<0.001)** | **12.90**  **(<0.001)** | **8.68**  **(<0.001)** | **10.14**  **(<0.001)** | 2.62  (0.344) | **4.38**  **(0.017)** | **4.91**  **(0.005)** | 3.17  (0.164) |
|  |  |  |  |  |  |  |  |  |  |  |  |

**TABLE S3** Mahalanobis distances among the eight southern Italian populations, associated to CVA in Figure 6.

|  | **SI1 *Barbus* lineage** | | |  | **SI2 *Barbus* lineage** | | | | |
| --- | --- | --- | --- | --- | --- | --- | --- | --- | --- |
| **ID pop** | A1 | A2 | A3 |  | A4 | A5 | T1 | T2 | T3 |
| A1 |  |  |  |  |  |  |  |  |  |
| A2 | 8.58 |  |  |  |  |  |  |  |  |
| A3 | 5.60 | 5.47 |  |  |  |  |  |  |  |
| A4 | 7.34 | 5.58 | 5.29 |  |  |  |  |  |  |
| A5 | 7.19 | 5.59 | 6.37 |  | 5.35 |  |  |  |  |
| T1 | 5.91 | 10.50 | 7.72 |  | 8.06 | 7.73 |  |  |  |
| T2 | 6.91 | 8.28 | 6.69 |  | 6.13 | 6.32 | 5.75 |  |  |
| T3 | 8.36 | 7.52 | 7.36 |  | 6.25 | 5.85 | 6.36 | 3.95 |  |

**Supporting Information FIGURES (S1-S3)**

*
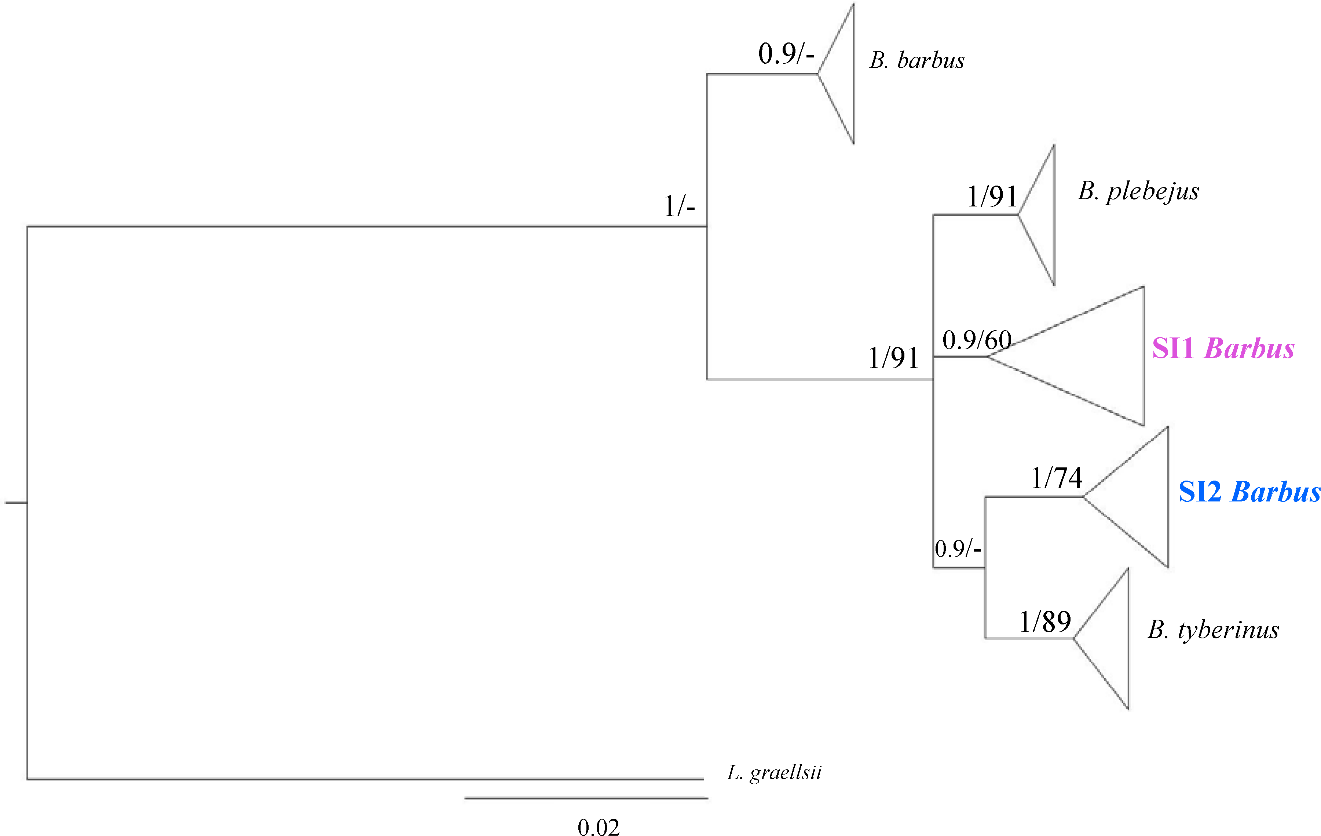
*

**FIGURE S1** D-loop phylogenetic tree built on 871 bp length haplotypes, produced in this study and retrieved from GenBank (*c.f.* Zaccara et al., 2019). Statistic support is given and expressed both as posterior probability and bootstrap values. The tree was rooted on *Luciobarbus graellsii* (GenBank accession number MG827110)*.*

**
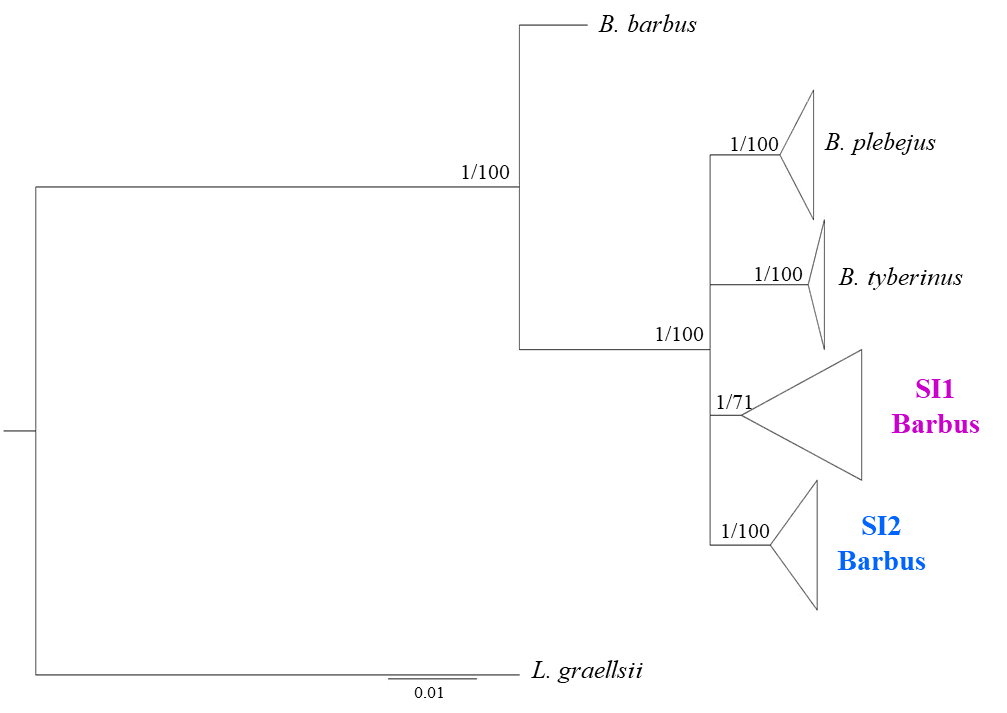
**

**FIGURE S2** Cyt *b* and D-loop combined phylogenetic tree built on 1599 bp length haplotypes, produced in this study and retrieved from GenBank (*c.f.* Zaccara et al., 2019). Statistic support is given and expressed both as posterior probability and bootstrap values. The tree was rooted on *Luciobarbus graellsii.*

*

*

**FIGURE S3** Mismatch distribution trends for SI1 **(A)** and SI2 **(B)** *Barbus* lineages. Solid lines represent the estimated trend expected under a model of sudden demographic expansion.
